# Effects of acute alcohol consumption on cardiac excitation, conduction, and repolarization: results from the Munich Beer Related Electrocardiogram Workup Study (MunichBREW)

**DOI:** 10.1007/s00392-021-01839-6

**Published:** 2021-03-18

**Authors:** Stefan Brunner, Cathrine Drobesch, Rebecca Herbel, Moritz F. Sinner

**Affiliations:** 1 Department of Medicine I, LMU Klinikum, University Hospital, LMU Munich, Marchioninistr. 15, 81377 Munich, Germany; 2 German Centre for Cardiovascular Research (DZHK), Partner Site: Munich Heart Alliance, Munich, Germany

Sirs:

Alcohol consumption may result in both deleterious and beneficial cardiovascular effects. Chronic heavy alcohol consumption can lead to systolic and diastolic dysfunction and left ventricular dilatation culminating in severe alcoholic cardiomyopathy [1]. Acute excessive alcohol intake has been repeatedly associated with the ‘Holiday Heart Syndrome’. It is characterized by the occurrence of supraventricular and ventricular arrhythmias in otherwise healthy individuals [2]. So far, one of the largest prospective study in this context is the MunichBREW study, investigating over 3000 voluntary participants at the 2015 Munich Octoberfest for cardiac arrhythmias under the influence of acute alcohol exposure. The study demonstrated a clear association of acute alcohol consumption with cardiac arrhythmias and sinus tachycardia in particular. As a potential trigger for cardiac arrhythmias emerged an imbalance of the autonomic nervous system as indicated by a significant reduction of respiratory sinus arrhythmia [3, 4].

In the MunichBREW study, electrocardiograms (ECGs) were analyzed qualitatively only [3]. Importantly, an analysis of quantitative ECG characteristics has not been performed yet. Here, we thus aimed to systematically investigate these quantitative ECG characteristics, representing cardiac excitation, conduction, and repolarization.

The MunichBREW study originally enrolled 3042 voluntary participants. After exclusions, 3,012 of them were included in the present analysis. Participants had to be ≥ 18 years old and provide written informed consent to study inclusion. Four individuals who presented with a breath alcohol concentration (BAC) ≥ 3.00 g/kg were excluded due to German law, and 26 individuals had uninterpretable ECGs. The ethics committee at the Ludwig Maximilians University of Munich, Germany approved the study, which was registered at clinicaltrials.org (NCT02550340).

In all study subjects, ECG recordings of 30 s duration were obtained using the smart phone-based AliveCor device (AliveCor, San Francisco, CA, USA). BAC in gram per kilogram (g/kg) was determined using a Dräger Alcotest 7510 handheld device (Drägerwerk AG, Lübeck, Germany). Clinical covariates age, sex, country of origin, history of heart disease, use of cardiovascular drugs, use of antiarrhythmic drugs, and active smoking status were collected by questionnaire. Quantitative ECG analysis included heart rate, PR interval, QRS duration, and QTc interval. QTc corrected for residuals of heart rate, age, and sex. All ECGs measurements were performed caliper-based on digital ECG recordings by two experienced cardiologists blinded to BAC and covariates. Results are expressed as mean ± standard deviation. We employed linear regression to test the association between quantitative ECG measures and BAC, adjusting for clinical covariates. We considered *p* < 0.05 statistically significant.

The mean age of the study cohort was 35.0 ± 13.2 years. Thirty percent of the participants were female. The participants originated from 60 different countries. The most reported nationalities were German (69%), US-American (5%), Austrian (4%), and Australian (4%). Six percent reported an underlying heart disease and 3% reported a known arrhythmia. Six percent used cardiac medication and 29% were active smokers.

ECG analysis revealed a mean heart rate across all participants of 91.0 ± 15.7 beats per minute (bpm) [median (25th; 75th): 90 (80;101) bpm]. Mean PR interval was 135.5 ± 27.0 ms [median (25th; 75th): 133 (117;151) ms], mean QRS duration was 115.2 ± 20.8 ms [median (25th; 75th): 113 (101;127) ms], and mean QTc interval was 369.0 ± 24.4 ms [median (25th; 75th): 368 (354;382) ms].

**Fig. 1:**
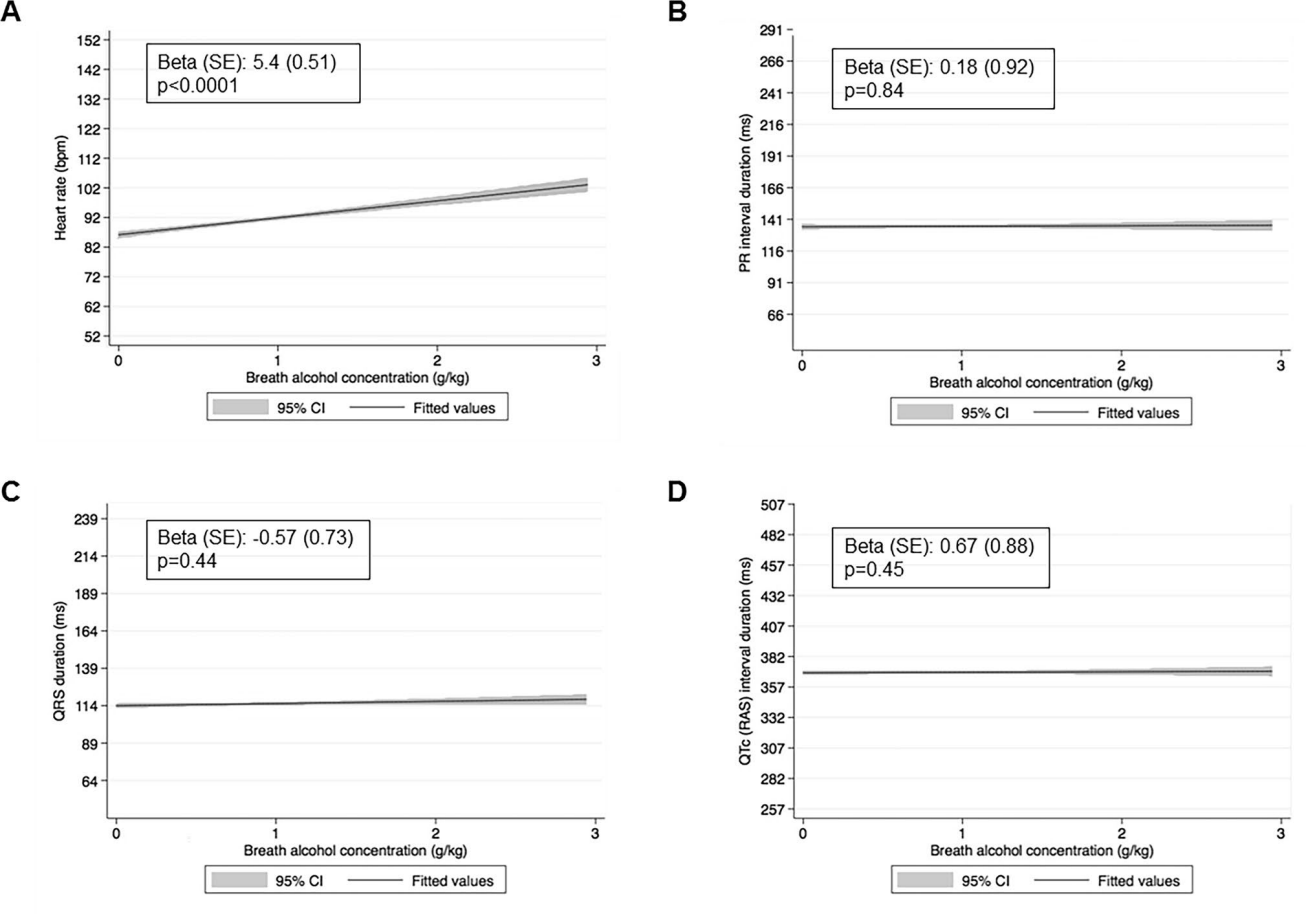
Regression lines of breath alcohol concentrations with **a** Heart rate, **b** PR interval, **c** QRS duration, and **d** QTc interval. In each panel, the solid lines illustrate the fitted regression, the shaded areas indicate the respective 95% confidence intervals around the regression lines. Insets report the effect size beta and its standard error (SE). RAS indicates the QT interval is corrected for rate, age, and sex

Regression analysis, adjusted for age, sex, country of origin, prevalent heart disease, known arrhythmias, cardiac medication use, and smoking status, showed a significant association of heart rate with increasing BAC [Beta (standard error (SE)): 5.4 (0.51); *p* < 0.001]. There was no significant association of increasing BAC and PR interval [1.8 (0.92); *p* = 0.84], QRS duration [− 0.57 (0.73); *p* = 0.44], and QTc interval [0.67 (0.88); *p* = 0.45] (Fig. 1).

In of one the largest prospective trials on the ECG effects of acute alcohol intake, we could show that quantitatively analyzable ECGs can be successfully obtained under unfavorable recording conditions at the Munich Octoberfest. As the main result, we clearly demonstrate a significant association with increasing heart rate and BAC. There was no association between alcohol and PR interval, QRS duration, and QTc interval.

The adverse cardiovascular effects following acute alcohol consumption may be related to the release of catecholamines, imbalance of the autonomic nervous system, electrophysiological consequences of alcohol metabolites, and deranged plasma electrolytes. A variety of ECG changes has been described in the setting of acute alcohol intoxication including a new onset of ventricular and atrial arrhythmias, referred to as the ‘Holiday Heart Syndrome’. Further, small case series described prolongation of the PR, QRS, and QT intervals, measures that reflect cardiac excitation, conduction and repolarization.[5–8]

In the previous, prospective MunichBREW trial, we observed a strong association of qualitatively assessed sinus tachycardia with increasing BAC under real-life conditions, which persisted after adjusting for confounders. Our current findings of quantitative analyses of ECG measures further confirm prior studies reporting an increase in heart rate following alcohol intake in experimental settings. The increase in heart rate may reflect an imbalance of the autonomic nervous system resulting from both an increase in sympathetic activity and a decrease of vagal tone [9, 10].

However, we did not observe an association of BAC with PR, QRS, and QTc intervals. This is in contrast to previous reports. Yet as the MunichBREW study is among the largest investigations in this field, it may be inferred that possible alcohol-induced alterations of cardiac conduction and repolarization may not be clinically relevant. In addition, previously reported effects may have been overestimated based on small study sizes. Alternatively, prior cohorts may have had a higher prevalence of underlying conditions, whereas our cohort was relatively young and healthy. An increased sympathetic tone may thus be the primary proarrhythmic mechanism in our cohort.

A number of considerations are warranted. The lively conditions at the Munich Octoberfest may have influenced the results. Particularly, the circumstances have precluded collecting a more detailed history and additional physical or technical evaluations. Further, with a single ECG recording, we cannot elucidate the temporal relation between alcohol consumption and the occurrence of ECG changes.

In conclusion, the so far largest prospective study associating quantitative ECG measures with acute alcohol exposure revealed a strong association between increasing BAC and heart rate. There was no significant association between PR, QRS, and QTc intervals and increasing BAC. Further studies are required to analyze the temporal relation of alcohol consumption and the occurrence of ECG changes.
